# Functional analysis of a novel *de novo SCN2A* variant in a patient with seizures refractory to oxcarbazepine

**DOI:** 10.3389/fnmol.2023.1159649

**Published:** 2023-04-20

**Authors:** Xiaoyue Hu, Miao Jing, Yanping Wang, Yanshan Liu, Ying Hua

**Affiliations:** ^1^Department of Neurology, Affiliated Children's Hospital of Jiangnan University (Wuxi Children's Hospital), Wuxi, China; ^2^Department of Pediatric Laboratory, Affiliated Children's Hospital of Jiangnan University (Wuxi Children's Hospital), Wuxi, China

**Keywords:** early-onset epileptic encephalopathy, *SCN2A*, oxcarbazepine, patch-clamp, precise medicine

## Abstract

**Objective:**

We admitted a female patient with infantile onset epilepsy (<3-month-old). The use of oxcarbazepine exacerbated epileptic seizures in the patient. In the present study, we aimed to identify the genetic basis of the infantile onset epilepsy in the patient, and determine the correlations among genotype, phenotype, and clinical drug response.

**Methods:**

We described the clinical characteristics of an infant with refractory epilepsy. Whole exome sequencing (WES) was used to screen for the pathogenic variant. Whole-cell patch-clamp was performed to determine functional outcomes of the variant.

**Results:**

WES identified a novel *de novo SCN2A* variant (c.468 G > C, p.K156N) in the patient. In comparison with wildtype, electrophysiology revealed that *SCN2A*-K156N variant in transfected cells demonstrated reduced sodium current density, delayed activation and accelerated inactivation process of Na^+^ channel, all of which suggested a loss-of-function (LOF) of Na_v_1.2 channel.

**Conclusion:**

We showed the importance of functional analysis for a *SCN2A* variant with unknown significance to determine pathogenicity, drug reactions, and genotype–phenotype correlations. For patients suffering from early infantile epilepsies, the use of oxcarbazepine in some *SCN2A*-related epilepsies requires vigilance to assess the possibility of epilepsy worsening.

## Introduction

The *SCN2A* gene is located on chromosome 2q24.3 and encodes the voltage-gated sodium channel (VGSC) Na_v_1.2. *SCN2A* variants are related to epileptic seizures, intellectual disability, autism spectrum disorders, and periodic ataxia (Suddaby et al., 2019; Reynolds et al., 2020). *SCN2A* variants could result in a wide spectrum of epilepsy, ranging from benign self-limited epilepsy to severe epileptic encephalopathy. Epileptic seizures in patients with *SCN2A* variants occur early, mostly in the neonatal period or at early infancy (Wolff et al., 2019). The Na_v_1.2 channel is expressed in the axon initial segment and Ranvier nodes of fetal myelinated nerve fibers, which may explain the major impact of *SCN2A* variants on fetal neural development and early-onset neurological diseases (Wolff et al., 2017).

Missense mutation is the most common type of mutations in *SCN2A* gene (Wolff et al., 2017; Zeng et al., 2022). Minor changes in amino acid sequence caused by missense variants may lead to different functional outcomes in sodium channels, including gain-of-function (GOF), loss-of-function (LOF), or mixed dysfunctional type (GOF/LOF; Hedrich et al., 2019). Previous studies have shown that hereditary variants mainly manifest as benign (familial) neonatal/infant epilepsy (Zara et al., 2013; Kim et al., 2020), whereas *de novo* variants mostly manifest as developmental and epileptic encephalopathy (DEE), such as Ohtahara syndrome, epilepsy of infancy with migrating focal seizures (EIMFS), West syndrome, Dravet syndrome, Lennox–Gastaut syndrome, and unclassifiable early-onset epileptic encephalopathy (EOEE; Wolff et al., 2017; Zeng et al., 2022). Patients with seizure onset at less than 3-month-old often carry the *SCN2A* GOF variants of the *SCN2A* gene (Wolff et al., 2017). Treatment of these patients with sodium channel blockers (SCBs), such as oxcarbazepine (OXC), carbamazepine (CBZ), phenytoin (PHT), and lamotrigine (LTG) can improve seizure outcome. However, when epileptic seizures occur in patients over 3-month-old, the identified *SCN2A* variants are usually LOF, and SCBs will aggravate epileptic seizure (Wolff et al., 2017; Brunklaus et al., 2020).

In the present study, we reported a case of EOEE associated with a novel *de novo SCN2A* variant (K156N). The patient developed seizures on the third day after birth that were frequent and refractory to drug treatment. Unlike the most cases reported in the literature, the patient’s seizures were aggravated after treatment with oxcarbazepine. Finally, the patient was seizure-free following the ketogenic diet. Further investigation was performed to determine the pathogenicity of the variant, and correlations among genotype, phenotype, and clinical drug response.

## Materials and methods

### Study participant and genetic analysis

The patient was screened for pathogenic variants through whole-exome sequencing (WES). Sanger sequencing was performed to verify the variants in the family. The pathogenicity of the variants was assessed according to the criteria recommended by the American College of Medical Genetics and Genomics (ACMG).

### Plasmid construction

The whole length of *SCN2A* coding sequences was synthesized and cloned into pcDNA3.1 (+) vector, which was linearized by Takara’s restriction enzymes (SnaBI and NheI). Then primers were designed to perform mutagenesis in the wild-type construct (Supplementary Table S1). Basically, PCR was used to amplify the wild-type *SCN2A* (NM_021007.3) gene fragment and the mutated *SCN2A* gene fragment, respectively. Meanwhile, 3xFLAG was fused at the C terminus of *SCN2A*, and ClonExpress MultiS One Step Cloning Kit (Vazyme) was used to ligate the above-purified gene fragments into the linearized vector by means of homologous recombination. Finally, Sanger sequencing was performed to verify the accuracy of the cloned *SCN2A* gene sequence and the induced variant.

### Cell culture and plasmid transfection

HEK-293 cells were inoculated into 6-well plates with DMEM medium of 10% fetal bovine serum and transfected with Lipofectamine 3000 kit (Invitrogen) for 48 h. Mutated Na_v_1.2 α-subunit and β-subunit were co-expressed.

### Electrophysiology

Extracellular fluid contains the following components: 140 mmol/L NaCl, 3.5 mmol/L KCl, 10 mmol/L D-Glucose, 10 mmol/L HEPES, 1 mmol/L MgCl_2_·6H_2_O, 2 mmol/L CaCl_2_·2H_2_O, and 1.25 mmol/L NaH_2_PO_4_·2H_2_O (pH 7.4, NaOH modulation). Intracellular fluid contains the following components: 20 mmol/L KCl, 115 mmol/L K-Aspartic, 5 mmol/L EGTA, 10 mmol/L HEPES, 1 mmol/L MgCl_2_·6H_2_O, and 2 mmol/L Na_2_-ATP (pH 7.2, KOH modulation). A coverslip lined with cells was placed in a recording chamber under an inverted microscope. During the experiment, the solutions were withdrawn from the chamber by a peristaltic pump. The whole cell voltage clamp mode was adopted, with a sampling frequency of 20 KHz and series resistance of approximately 2 ± 0.5 MΩ. A total of 70% resistance and capacitance compensation was provided. Experimental data was collected by HEKA (HEKA Elektronik Dr. Schulze GmbH, Lambrecht, Germany) amplifiers.

### Data analysis and graphing

Electrophysiological kinetic plan for sodium channels was performed as follows:

1. The recording procedures for the I-V curve included the holding voltage set at −120 mV for 200 ms and stepped every 5 mV to gradually increase from −100 mV to +90 mV. Following 50 ms, the voltage was returned to −120 mV. An IV curve was plotted with current density (pA/pF) as the vertical coordinate.
2. The recording procedures for the activation curve included the holding voltage set at −120 mV for 200 ms and stepped every 5 mV to gradually increase from −100 to +90 mV. Following 50 ms, the voltage was returned to −120 mV. The Boltzman equation was used to fit the activation curve and determine the pulse voltage (V_1/2_) and slope factor (k) when the channel activation reached 50%. The curve was plotted with the conductance G/Gmax as the vertical coordinate. The membrane potential ranged between −100 and 0 mV.
3. The recording procedures for the deactivation curve included the holding voltage set at −120 mV for 200 ms and stepped every 5 mV to gradually increase from −130 to −10 mV, and this voltage was maintained for 1,000 ms. Then, the test voltage was set to −10 mV for 50 ms, and restored to −120 mV. The Boltzmann equation was used to fit the deactivation curve and determine the pulse voltage (V_1/2_) and the slope factor (k) upon the channel deactivation of 50%. The curve was plotted with the current I/Imax as the vertical coordinate.
4. The recording procedures for the recovery curve included the holding voltage set at −120 mV with double-pulse stimulation. First, −10 mV pre-voltage was set and maintained for 50 ms to deactivate the sodium current, and then stepped to −120 mV for different time periods to restore the sodium current. Next, the test voltage was set to −10 mV for 50 ms to test the current after the restoration, and then restored to −120 mV. The recovery curve was plotted with the current Itest/Ipre as the vertical coordinate, and the recovery time ranged 0–30 ms. The recovery time constant (τ_rec_) of the sodium channel was obtained by fitting the curve with the exponential equation.

### Statistical analysis

All results are expressed as the mean ± standard error of the mean (m ± SEM). Statistical significance between the mutant type and wild type were calculated using unpaired Student’s t-test. A significance level of *p* < 0.05 was considered statistically significant. Data were analyzed by IBM SPSS 26.0 (SPSS, Inc., Chicago, IL, United States) software and GraphPad Prism 8.0 software (GraphPad Software, La Jolla, CA, United States).

## Results

### Clinical characteristics of the patient

**Figure 1:**
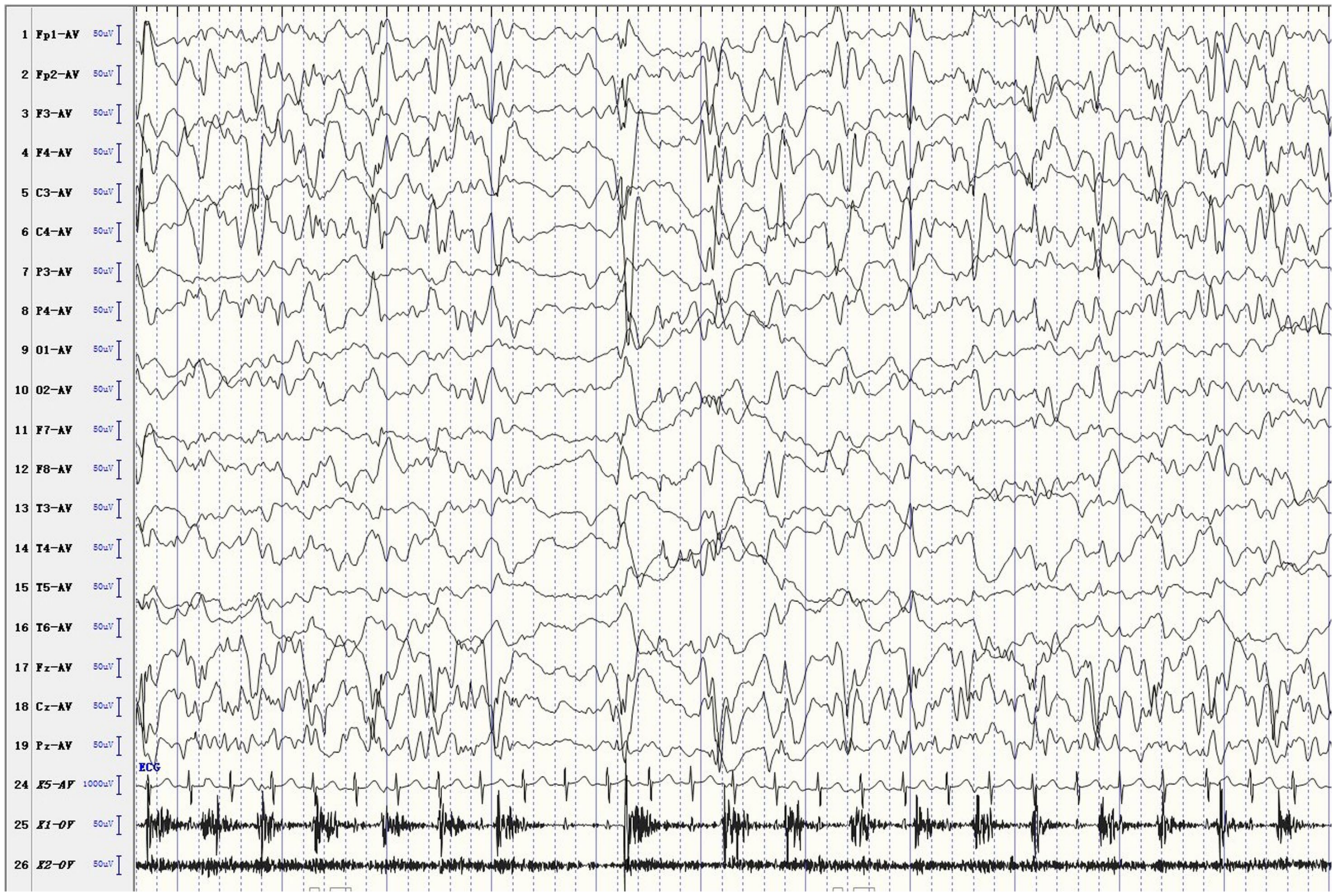
Ictal EEG. The patient developed rhythmic jitter of the left limb, and EEG showed mixed fast and slow waves and sharp waves in the right hemisphere.

The female patient was delivered by cesarean section at full term, without medical records of birth injury, asphyxia, or special family history. Her mother was healthy during pregnancy. She developed convulsions 3 days after birth and was diagnosed with neonatal convulsions that were treated with oral phenobarbital. One month and 3 days after birth, she had a second episode of cluster tics that lasted for up to 1 min. Cranial MRI did not show significant abnormalities. Routine blood test, blood biochemistry, as well as blood and urine metabolic screening indicated no abnormalities. Video electroencephalogram (EEG) showed multifocal sharp waves (Figure 1). Levetiracetam (LEV) was given orally to control epileptic seizures, but the effect was poor. Twenty days later, the patient had recurrent episodes of clustered focal seizures that occurred several times a day. She was then treated with oxcarbazepine (OXC) afterwards. However, the frequency of episodes increased to dozens of seizures a day after OXC treatment. OXC was discontinued and replaced by topiramate (TPM) combined with micropump infusion of midazolam for stopping the seizures but resulted in no effect. Three days later, the patient still had frequent convulsions, thus propofol and sodium valproate (VPA) injection were added, but neither one produced any effect. The ketogenic diet was introduced eventually, and the frequency of seizures gradually decreased. Within 1 week after initiating the ketogenic diet, the patient was seizure-free. Currently, the patient remains seizure free, without recurrence of epilepsy. But she still has delayed intellectual and motor development. At 1 year and 8 months old, she has been able to walk alone and say “Mom, Dad.” Meanwhile, she has demonstrated ataxia, with the manifestation of paroxysmal gait instability and slurred speech.

### A novel *de novo* variant in the *SCN2A* was identified *via* WES

**Figure 2:**
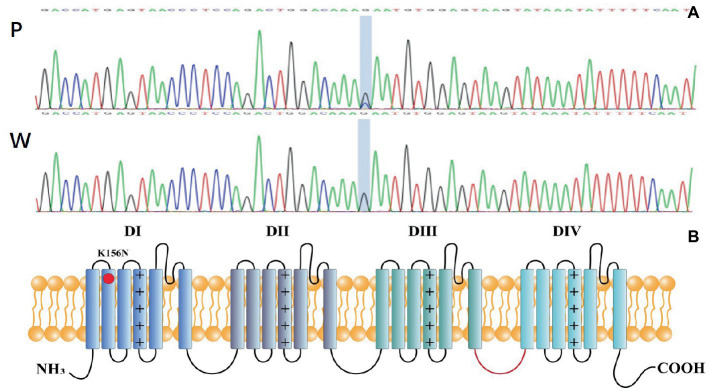
**(A)** P: Mutation genotype of the patient c.468G > C (p.K156N)，W: wildtype genotype of the patient’s parents. **(B)** Topology diagram of the human Na_v_1.2 channel’s α subunit. The location of the K156N variant described in the study is shown by a red circle.

**Figure 3:**
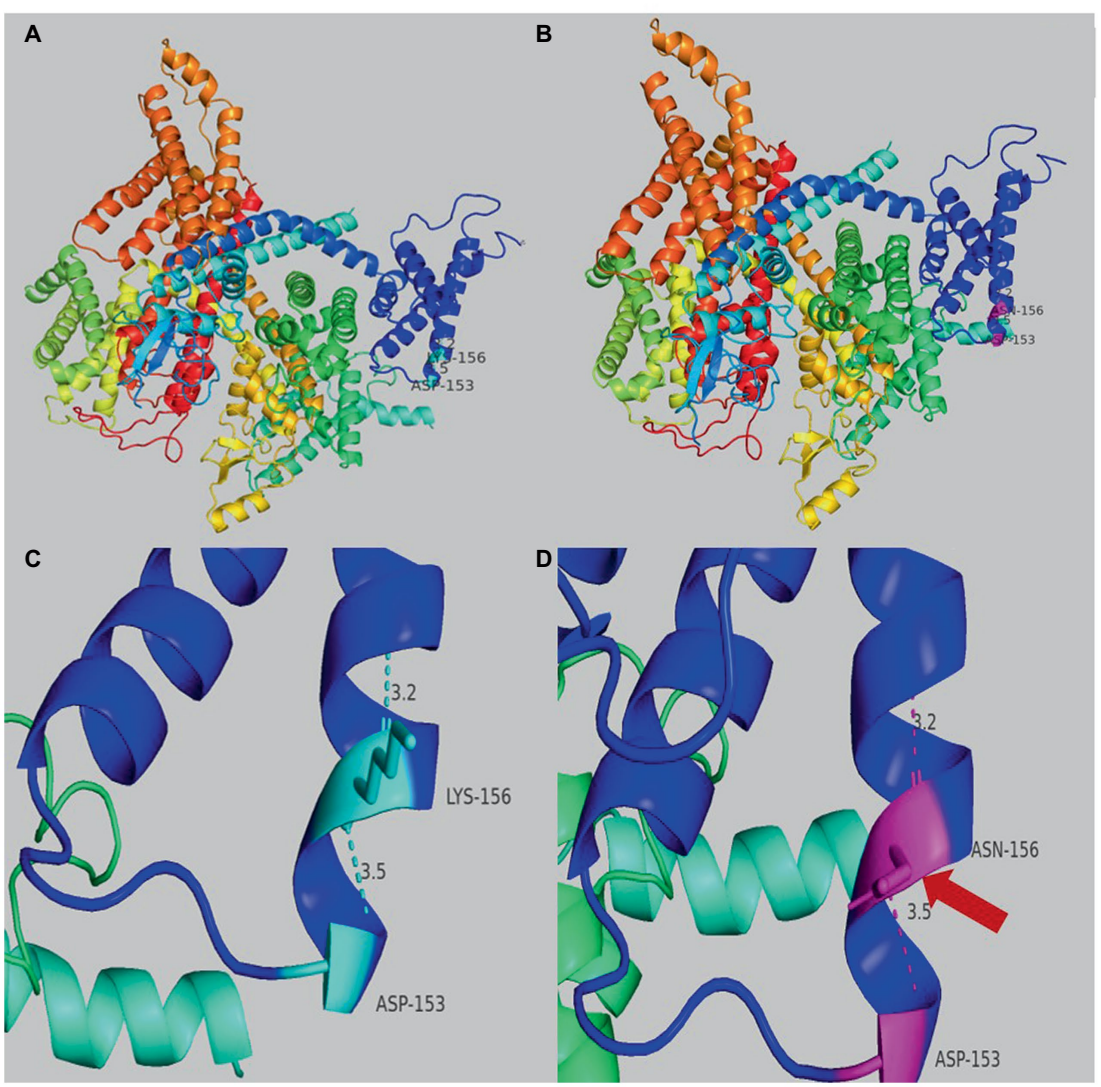
**(A)** Overview of the three-dimensional structure of the *SCN2A* wild-type protein. **(B)** Overview of the three-dimensional structure of the *SCN2A* K156N variant protein. **(C)** Details structure of the *SCN2A* wild-type protein around the mutated site. **(D)** Details of the *SCN2A* K156N variant protein around the mutated site. The red arrow indicates the difference between wild-type and variant protein residues.

Genetic testing suggested a *de novo* missense variant in the *SCN2A* gene (NM_021007.3) c.468G > C (p.K156N; Figure 2A). The variant has neither been previously reported in the literature, nor included in the disease variant database and public population databases, such as ClinVar, HGMD and gnomAD. Multiple software have predicted that the variant tends to be deleterious (REVEL = 0.81). It is predicted to be likely pathogenic (PS2, PM2, PM5, PP2, PP3) according to ACMG guidelines. The variant is located in the transmembrane structure, fragment 2 of structural domain I (Figure 2B). Three-dimensional structure stimulation suggested that this variant did not cause significant changes to the conformation of the protein (Figures 3A,B) except for a slight difference in the residues (Figures 3C,D).

### Electrophysiology results suggested K156N resulted in loss-of-function of Na_v_1.2 channel

**Figure 4:**
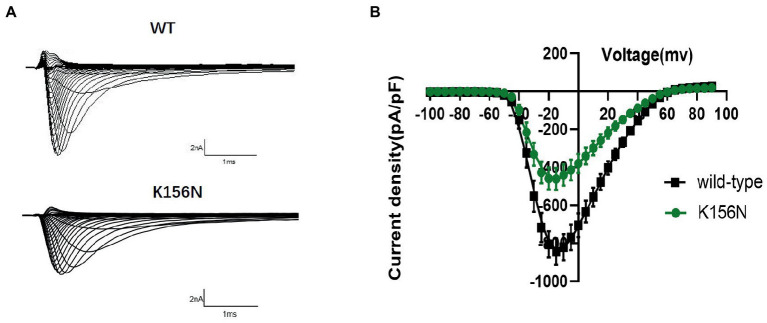
**(A)** Representative current recordings from WT and K156N Na_v_1.2 obtained using the voltage protocol shown in Figure 5B. **(B)** Current density for Na_v_1.2 wild-type(*n* = 15) and K156N variant(*n* = 15).

**Figure 5:**
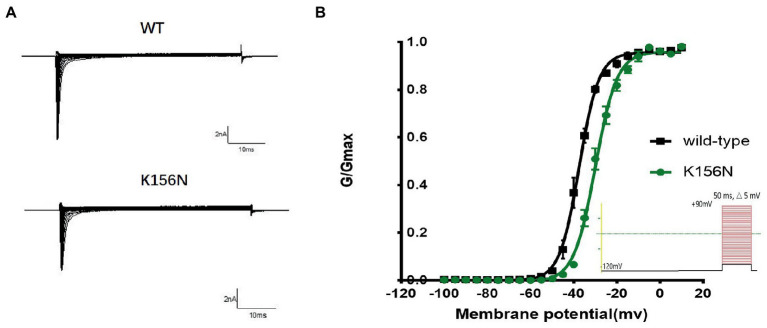
**(A)** Representative current recordings. **(B)** Voltage dependence of channels activation for wild-type (*n* = 15) and K156N variant (*n* = 15) using the voltage protocol shown as an inset.

**Figure 6:**
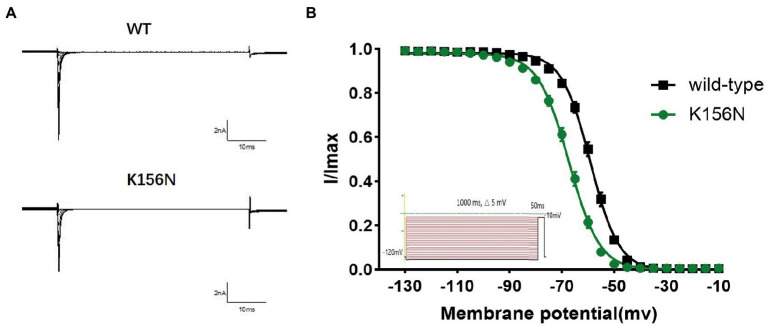
**(A)** Representative current recordings. **(B)** Voltage dependence of fast inactivation for wild-type(*n* = 15) and K156N variant(*n* = 15) channels using the voltage protocol shown as an inset.

**Figure 7:**
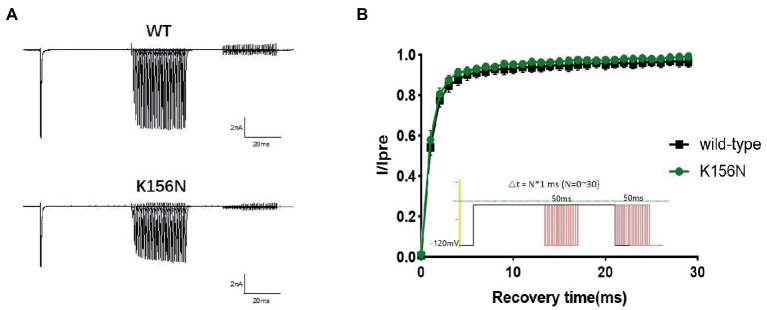
**(A)** Representative current recordings. **(B)** Time-dependent recovery from steady state inactivation for wild-type(*n* = 15) and K156N variant(*n* = 15) using the voltage protocol shown as an inset.

To determine the functional outcomes of the variant, we performed whole-cell patch-clamp electrophysiology. The current density-voltage relationship showed that the cells harboring K156N channels exhibited a current density of −459.72 ± 58.13 pA/pF, significantly reduced compared to −805.89 ± 69.75 pA/pF for wild-type channels (Figure 4; Table 1; *p* < 0.05). Figure 5 shows the voltage dependence of activation curves. In contrast to wild-type channels (−37.87 ± 0.86 mV), the half-maximal activation potential (V_1/2_) of K156N (−30.02 ± 0.85 mV) shifted by ∼7 mV in the depolarized direction (Table 1; *p* < 0.01). The steady-state inactivation curves are shown in Figure 6. The fast-inactivation curve of K156N channels exhibited a ∼ 8 mV hyperpolarizing shift of V_1/2_ (−67.29 ± 0.81 mV) as compared to wild-type (−59.16 ± 0.73 mV; see Table 1; *p* < 0.01). The slope factor K of the curves of wild-type and K156N variant were − 5.17 ± 0.11 and − 5.55 ± 0.10, respectively (see Table 1; *p* < 0.05). The fast-inactivation recovery time constant τ_rec_ obtained for K156N (11.92 ± 0.42 ms) did not differ significantly from that for wild-type (12.93 ± 0.48 ms; Figure 7; Table 1; *p* > 0.05). These results revealed that K156N variant induced LOF change of the Na_v_1.2 channel.

**Table 1:** Biophysical parameters of Na_v_1.2 wild-type and K156N variant.

	Peak current density	Voltage dep. of steady-state activation		Voltage dep. of steady-state inactivation		Recovery from inactivation
	Mean peak amplitude (pA/pF)	V_1/2_ (mV)	k	V_1/2_ (mV)	k	τ_rec_(ms)
Wild-type (*n* = 15)	−805.89 ± 69.75	−37.87 ± 0.86	3.51 ± 0.38	−59.16 ± 0.73	−5.17 ± 0.11	12.93 ± 0.48
K156N (*n* = 15)	−459.72 ± 58.13^*^	−30.02 ± 0.85^**^	4.37 ± 0.17	−67.29 ± 0.81^**^	−5.55 ± 0.10^*^	11.92 ± 0.42

Data are presented as means ± SEM, τ_rec_ is time constant, ^∗^*p* < 0.05, ^∗∗^*p* < 0.001.

## Discussion

### Phenotype of EOEE patients

The patient had focal paroxysmal seizures within the first 3 days after birth. EEG showed widespread multifocal discharges, and several anti-seizure medications (ASMs) were ineffective. Finally, epileptic seizures stopped after ketogenic diet therapy. She showed delayed development after seizure control and ataxia later in life, which is consistent with the diagnosis of early-onset epileptic encephalopathy due to variants in *SCN2A* gene. EOEE usually exists as refractory epilepsy during the neonatal period, with 20%–40% of cases diagnosed within approximately 3 months after birth (Ben-Shalom et al., 2017; Wolff et al., 2017). EOEE displays a variety of seizure types, including focal seizures, spastic seizures, tonic seizures, generalized tonic–clonic seizures, atonic seizures, and myoclonic seizures (Shi et al., 2012; Wolff et al., 2017). Focal seizures and spastic seizures are the most common, and focal seizures tend to be clustered. With the widespread use of next-generation sequencing technologies in clinical practice, multiple *SCN2A* variants have been reported in patients with severe EOEE, most of which are *de novo* (Wolff et al., 2017). Patients with *de novo* variants have more severe phenotypes, such as delayed intellectual and psychomotor development. Patients with developmental delays show little improvement even after epilepsy is controlled, further suggesting that *de novo* variants may have a significant impact on brain development. As a patient grows, the function of Na_v_1.2 was gradually replaced by Na_v_1.6 encoded by *SCN8A*. At that time, the *SCN2A* gene is predominantly expressed in cerebellar granule cells and unmyelinated nerve fibers (Vacher et al., 2008), which explains why our patient had neonatal or infantile epilepsy early in life and then developed ataxia later. These phenotypes are similar to reports from other *SCN2A* variants, such as A263V, R1883G, and S987I (Schwarz et al., 2016, 2019).

### Properties of the *SCN2A* variant

In the present study, the patient had a novel missense variant of the *SCN2A* gene, namely K156N. Zeng et al. (2022) reported another amino acid variant at the same locus in *SCN2A*, K156Q, which had a phenotype similar to our case and was diagnosed as EOEE. However, the patient’s seizures remained uncontrolled at follow-up until the age of 1 year and 4 months, and the use of ASMs was unknown (Zeng et al., 2022).

Na_v_1.2 is a pseudotetrameric protein that consists of four highly similar structural domains (I, II, III, IV). Each structural domain contains six transmembrane fragments from S1 to S6, of which S1 to S4 form a voltage-sensitive structural domain. S4 constitutes a voltage sensor sensitive to the difference in charge between the intracellular and extracellular sides of the membrane, and S5 to S6 form a pore loop and DEKA-selective filter (de Lera Ruiz and Kraus, 2015). Several disease-related hotspot variants in Na_v_1.2 are centered on S4 and S5 in the voltage-sensitive domain, the intracellular N- and C-terminal domains, and the pore loop around the ion-selective filter (Sanders et al., 2018). K156 is located in the S2 transmembrane region of DI, and variants in this transmembrane region have rarely been reported. Previous studies have suggested that over 80% of the *SCN2A* variants detected in patients with developmental retardation are located in the transmembrane region (Zeng et al., 2022). In consistent with mild phenotype of the patient, the three-dimensional protein model of the variant suggested that the changes were only observed in amino acid residues, which delivered little overall effect on the protein structure.

### Unfavorable oxcarbazepine treatment and functional study

This case is similar to some patients with Dravet syndrome. The patient had focal epileptic seizures, but the attacks were more frequent and did not show the feature of fever-sensitivity. Both *SCN2A* and *SCN1A* variants are responsible for Dravet syndrome. Different from *SCN1A* LOF variants, *SCN2A* GOF variants can also cause Dravet syndrome, and oxcarbazepine treatment is usually effective (Zeng et al., 2022).

For infants with epileptic seizures within 3 months after birth, SCBs, such as OXC, PHT, and CBZ are more effective, as the function of the variation of *SCN2A* is considered to be more likely to be GOF (Adney et al., 2020). Therefore, we selected OXC for treatment based on the genetic test results, but the result was contrary to our expectation. The patient’s seizures worsened with OXC. A study by Zeng et al. (2022) revealed that only 27% of patients with epileptic seizures within 3 months after birth were controlled by OXC. In addition, OXC resulted in exacerbation of seizures in three other patients (< 3-month-old) carrying three *SCN2A* variants, V424A, K1508I, and G1645R, respectively. Electrophysiology experiments in the present case showed a decrease of current density, slowed activation process, and accelerated inactivation process of the K156N variant. This finding suggested that the K156N variant presented a loss of channel function, which was considered to be the cause of seizure exacerbation after the administration of OXC.

Like *SCN1A* LOF variants in Dravet syndrome, recent studies described the expression of Na_V_1.2 channels in inhibitory neurons, indicating the mechanism of epilepsy due to *SCN2A* loss-of-function variants could also be the imbalance of excitatory/inhibitory neurons. A knockin mouse model carrying a patient-derived nonsense *SCN2A* variant resulted in absence-like seizures at 6–11 weeks of age. Further investigations suggested that LOF variants in *SCN2A* could reduce the excitability of inhibitory neurons expressing Na_V_1.2 and thus lead to epileptic seizures (Ogiwara et al., 2018). Besides, *SCN2A* loss-of-function promotes seizure by preventing potassium channels from properly repolarizing neurons between action potentials (APs), advancing the timing of subsequent APs, thus increasing the overall excitability (Spratt et al., 2021).

### Promising effect of ketogenic diet in treating epilepsy patients less than 3-month-old

In the present study, our patient had refractory epilepsy, but eventually achieved seizure-free immediately within 1 week after the application of ketogenic diet. A follow-up of over 1 year and 6 months showed that the patient had manageable seizure control. A higher percentage of patients less than 3-month-old with variants in the *SCN2A* gene have shown that ketogenic diet therapy is effective compared with patients over 3-month-old (Wolff et al., 2017; Su et al., 2018; Turkdogan et al., 2019; Kim et al., 2020; Miao et al., 2020; Tian et al., 2021). Turkdogan et al. (2019) reported a patient with a M136I variant that was diagnosed with Ohtahara syndrome. The patient was treated with ketogenic diet on the 39th day after birth and became seizure-free. Su et al. (2018) reported a patient with a W191C variant that initially presented with focal seizures and evolved into infantile spastic epilepsy on the third day after birth. Treatment with OXC and PHT did not show significant improvement in seizures. However, the patient achieved seizure-free after the application of the ketogenic diet. Ketogenic diet can also increase the threshold of convulsion in the Dravet syndrome mouse model with *SCN1A* variant, and reduce the convulsion attack (Dutton et al., 2011); Using ketogenic diet can inhibit the transmission of glutamate and activate ATP-sensitive K^+^ channel, reduce the transmission of chromosome junction and the excitability of neurons (Simeone et al., 2017). These maybe explain the anti-seizure mechanisms of ketogenic diet in *SCN2A* variants.

In conclusion, we described a case of an infant with EOEE due to a missense variant in the *SCN2A* gene. The patient was diagnosed with drug-refractory epilepsy due to epileptic seizures within 3 months after birth. The patient suffered an increase in frequency of epileptic seizures following the application of OXC. Finally, she became seizure-free after the ketogenic diet. Functional analysis showed a LOF *SCN2A* variant suggesting that some <3-month-old patients with epileptic seizures may not all exhibit GOF. Therefore, we still need to be alerted to the possibility of seizures worsening upon the application of SCBs.

This analysis has several limitations. We did not use sodium channel openers to further verify whether the mutant effect can be remedied. The effective mechanism of ketogenic diet treatment also needs further study.

## Data availability statement

The raw data supporting the conclusions of this article will be made available by the authors, without undue reservation.

## Ethics statement

The studies involving human participants were reviewed and approved by Ethics Committee of Wuxi Children’s Hospital. Written informed consent to participate in this study was provided by the participants' legal guardian/next of kin. Written informed consent was obtained from the individual(s) for the publication of any potentially identifiable images or data included in this article.

## Author contributions

XH and YL were the major contributors in writing the manuscript. XH, YW, and YH made a follow-up to the patient. XH and YL did plasmid construction, cell culture, and plasmid transfection. XH, YH, and MJ did electrophysiological test and data analysis. YH did graphing and the EEG monitoring work. All authors contributed to the article and approved the submitted version.

## Funding

This work was supported by Wuxi Taihu Lake Talent Plan Top Talents Project (grant no. HB2020086), Young project of Wuxi Health Committee (grant no. Q202258), the National Natural Science Foundation of China (grant no. 82070987), and Youth Foundation of Jiangsu Natural Science Foundation (grant no. BK20190599).

## Conflict of interest

The authors declare that the research was conducted in the absence of any commercial or financial relationships that could be construed as a potential conflict of interest.

## Publisher’s note

All claims expressed in this article are solely those of the authors and do not necessarily represent those of their affiliated organizations, or those of the publisher, the editors and the reviewers. Any product that may be evaluated in this article, or claim that may be made by its manufacturer, is not guaranteed or endorsed by the publisher.
